# “A good little tool to get to know yourself a bit better”: a qualitative study on users’ experiences of app-supported menstrual tracking in Europe

**DOI:** 10.1186/s12889-019-7549-8

**Published:** 2019-09-03

**Authors:** Johanna Levy, Nuria Romo-Avilés

**Affiliations:** 1 Institute of Women’s and Gender Studies, University of Granada, Granada, Spain; 2 Department of Social and Cultural Anthropology, University of Granada, Granada, Spain

**Keywords:** Menstrual apps, Period-tracking, Health-tracking, Qualitative study, Smartphone, Empowerment, User perception

## Abstract

**Background:**

Menstrual apps facilitate observation and analysis of menstrual cycles and associated factors through the collection and interpretation of data entered by users. As a subgroup of health-related apps, menstrual apps form part of one of the most dynamic and rapidly growing developments in biomedicine and health care. However, despite their popularity, qualitative research on how people engaging in period-tracking use and experience these apps remains scarce.

**Methods:**

Between June 2016 and March 2017, we conducted 26 qualitative interviews with menstrual app users living in Austria and Spain. The participants were asked about their practices and experiences regarding app-supported menstrual tracking. The interviews were audio recorded, transcribed verbatim, and coded using the software NVivo.

**Results:**

An inductive content analysis was performed and eight characteristics of app-supported menstrual tracking were identified: 1) tracking menstrual cycle dates and regularities, 2) preparing for upcoming periods, 3) getting to know menstrual cycles and bodies, 4) verifying menstrual experiences and sensations, 5) informing healthcare professionals, 6) tracking health, 7) contraception and seeking pregnancy, and 8) changes in tracking. Our study finds that period-tracking via apps has the potential to be an empowering practice as it helps users to be more aware of their menstrual cycles and health and to gain new knowledge. However, we also show that menstrual tracking can have negative consequences as it leads to distress in some cases, to privacy issues, and the work it requires can result in cessation. Finally, we present practical implications for healthcare providers and app developers.

**Conclusions:**

This qualitative study gives insight into users’ practices and experiences of app-supported menstrual tracking. The results provide information for researchers, health care providers and app designers about the implications of app-supported period-tracking and describe opportunities for patient-doctor interactions as well as for further development of menstrual apps.

## Background

### Health apps

In 2017, more than 325.000 health-related applications (called health apps hereafter) were available in the major app stores [1]. Given their high number and current annual growth rate of about 25%, health apps represent one of the most dynamic and rapidly growing technological developments in the areas of biomedicine and health care. Health apps cover a broad spectrum of functions ranging from providing general medical information to disease-specific apps and health and fitness apps. They address a broad audience including healthcare providers, medical students, patients, and the general public [2]. As their popularity has risen, so has research interest in health apps increased in recent years. Medical and public health studies adopting quantitative as well as qualitative approaches have examined health apps’ effectiveness regarding their ability to induce changes in health behavior [3–6], users’ perceptions of app-supported health-tracking have been analyzed with a focus on usability, acceptance and users’ opinions on design [7, 8], and other studies have addressed issues of user safety and identified risks associated with health-tracking [9, 10]. While most literature has focused on the potential of mobile digital devices in terms of health promotion and patient empowerment, some scholars have adopted more critical perspectives addressing issues such as shifts in responsibility, body enhancement and healthism (e.g. [11]).

### Menstrual apps

Mobile apps for menstrual-cycle tracking (hereafter termed as menstrual apps or period-tracking apps), the focus of this paper, represent a subgroup of health apps. Menstrual apps enable the observation and analysis of menstrual cycles and a variety of related factors. The home screens of most period-tracking apps show a numerical countdown or a graphic illustration indicating the number of days until the start of the next period or ovulation. Most menstrual apps offer three additional functions: 1) the tracking of menstrual cycle-associated factors such as mood swings, pain, sleeping patterns, intake of medication and contraceptives, sex life, vaginal discharge, food cravings and exercise; 2) a menstrual calendar where period and ovulation dates as well as days on which additional data have been entered by users are highlighted in specific ways; and 3) an analysis screen with graphs, tables or numerical depictions that provide users with statistical information such as average cycle lengths or changes in body weight, mood, body temperature etc.. A few menstrual apps offer access to online forums, some provide medical information via links and pop-up windows, and several facilitate user interaction with healthcare professionals, other users or their intimate partners.

In 2016, the download volume for menstrual apps was estimated to have reached 200 million [12]. However, despite their popularity, limited scholarly attention has been paid to app-supported menstrual tracking so far. Among the existing research on menstrual apps, there are some medical and computer science studies addressing the design and performance of pregnancy-related and fertility-tracking apps [13–15], one investigation evaluating patient satisfaction and compliance with mobile app reporting on heavy menstrual bleeding [16], as well as one study drawing on menstrual and ovulation records of a mobile phone app in order to examine the relationship between menstrual cycles and the timing of ovulation and with the aim of contributing to the improvement of menstrual apps’ performance [17].

In addition, Epstein et al. released a study in 2017 which reports on practices of menstrual tracking in the US context highlighting the principal reasons why women track their cycles [18]. There are studies on pregnancy and parenting apps which examine users’ reasons for and expectations of engaging in self-tracking during pregnancy and early parenthood [19, 20]. In a content analysis of sex and fertility tracking apps, Lupton highlights how the apps’ depictions of bodies reinforce normative gender stereotypes [21]. Underlying gendered assumptions do not go unnoticed by users as has been shown in a paper published by the lead author regarding users’ perception of menstrual apps [22]. The present paper focuses specifically on app-supported menstrual tracking in the European context. Given that most of the research carried out on menstrual tracking does not provide a detailed analysis of users’ practices and experiences, our aim is to contribute to filling this gap and to identify the implications of period-tracking via apps. Following the description of the methods, we present eight characteristics of app-supported menstrual tracking. In the discussion section, the findings will be presented within the framework of related literature. Against the background of techno-utopian accounts on the one hand and more skeptical perspectives on the other, we examine the role of period-tracking apps in user empowerment and menstrual health literacy. Finally, we present suggestions for health professionals and app designers.

## Methods

Between June 2016 and March 2017, we conducted a qualitative interview study on the experiences of and responses to app-supported menstrual tracking with participants living in Austria and Spain. Following the transcription of the interview recordings, a content analysis was performed through the general reading of the transcripts and inductive codification using the software NVivo. We then turned to related literature to frame the themes we had identified. In order to adhere to qualitative reporting standards, we followed the 32-item consolidated criteria for reporting qualitative studies (COREQ) checklist [23].

### Participants

The likelihood of adopting health apps has been shown to depend strongly on sociodemographic characteristics: in the European context, a study carried out with Dutch participants noted that app users were generally younger with higher levels of education and e-health literacy than non-users [24]. Similarly, an online survey conducted in the Czech Republic found that app users reported more frequent smartphone use, more expert phone skills and that they were more likely to be female [25]. Given the limited number of studies reporting on menstrual app use and with the aim of drawing on a diverse pool of participants, inclusion criteria for our study were: being over 18 years of age and the use or former use of an app for menstrual tracking. There were 26 participants in total: eleven living in Vienna, Austria or the surrounding area; thirteen from the province of Granada province in the south of Spain; and to maximize the variation of participants’ profiles and experiences, two additional interviews were performed, with one participant from Bologna, Italy and one from San Sebastian, Spain. Three participants had stopped tracking their menstrual cycles via apps prior to the interviews, while the remaining 23 were using either one or several period-tracking apps (several (7) of our participants mentioned using or having used more than one menstrual app at a time and three users reported to having used different period-tracking apps before). Three brands were more popular than the other period-tracking apps as they were used or had been used by the majority of our participants (22). All the other apps (8) were used by only one interviewee each. Most of the participants in our study reported long-term engagement in app-supported menstrual tracking: at the time of the study, more than two thirds of the participants (17) had used their menstrual apps for one to three years. Six interviewees stated that they had been tracking their periods for six months or less and one person estimated having started using a menstrual app at least five years ago. Three of the participants were formerly known to the interviewer and one was a colleague of the second author. Regarding their gender identity and sexual orientation, all but one interviewee identified as female, and twenty participants were heterosexual. One interviewee self-identified as non-white. The age range, level of education and digital literacy of our participants confirmed the findings of the above-mentioned studies [24, 25]: our participants’ ages ranged from 18 to 40 years old with 21 being under the age of 34. Most were students or planning to study in the near future (22), and four held a PhD. Most (23) self-identified as users with average (11) or high (12) digital media literacy. Five stated that they suffer from a medical condition that influenced their menstrual cycles. Table 1 summarizes the demographic information and app usage of the participants.

**Table 1:** Participant demographics and menstrual app use

Gender		
Female	25	96%
Non-binary	1	4%
Age		
18–25 years old	8	31%
26–33 years old	13	50%
34–40 years old	5	19%
Race		
white	25	96%
non-white	1	4%
City/Region, Country		
Vienna, Austria	11	42%
Granada Province, Spain	13	50%
San Sebastian, Spain	1	4%
Bologna, Italy	1	4%
Sexual Orientation		
heterosexual	20	77%
homosexual	4	15%
bisexual	2	8%
Health Status		
condition affecting menstrual cycle	5	19%
Education		
apprenticeship	4	15%
high school diploma	4	15%
bachelor’s or master’s degree	14	54%
PhD	4	15%
Digital Literacy^a^		
basic	2	8%
average	11	42%
high	12	46%
Menstrual App Use		
user	23	88%
former user	3	12%
Length of Menstrual App Use^b^		
< 6 months	6	23%
1–3 years	17	65%
> 3 years	1	4%

^a^one participant did not disclose digital literacy

^b^two participants did not disclose length of use

Regarding the interviewees’ motivations for participating in the study, it was interesting that several expressed a wish to fight against menstrual stigma:

“I think it is an incredibly important topic, which should be definitely spoken about. I think this is the reason why we are here today (laughter). No, I really think it is important and nobody should be ashamed or talk it down somehow.” (Pt.1, 29 years old).

While some participants simply seemed to want to help, others pointed out that the interviews were an opportunity to learn more about their cycles and menstrual apps:

“So I am currently [learning about my cycle], this is also why I immediately, I immediately wanted to participate in the study” (Pt.5, 37 years old).

### Procedure

The ethics committee of the University of Granada approved the study. In a purposive sampling procedure, interview participants were recruited via the authors’ private and professional networks, social media platforms and through the contacting of health centers and non-governmental organizations (NGOs). As we are from Vienna, Austria and Granada, Spain, we decided to focus on these two regions. All the interviews were carried out by the lead author of the paper, a female PhD student at the University of Granada trained in Molecular Biology and Gender Studies. The second author, an experienced researcher and professor of Social Anthropology at the University of Granada, supervised the process. First sample interviews were conducted in Vienna, Austria in June and July 2016. A second round of interviews with participants from Vienna was carried out in September 2016, followed by the recruitment and interviewing of menstrual app users in Granada, Spain between October 2016 and March 2017. The interviews with the two participants from Bologna, Italy and San Sebastian, Spain were completed in February and March 2017. All but one interview were conducted in the native language of the participants (Spanish or German). One was completed in English. Four interviews were carried out via online conference tools, while the remaining 22 interviews were conducted face-to-face. The interview venues were chosen by the interviewees and took place at their homes and workplaces or in cafés. The interviews lasted between 45 min and one hour and participants were encouraged to open their period-tracking apps and go through their functions during the interview. All interviews were audio-recorded with participant consent and transcribed for analysis. After each interview, participants completed a short questionnaire designed by the authors to gather socio-demographic data. The interviews were supplemented by field notes taken after the interviews. The process of data gathering was terminated when the variety of profiles sought had been completed and data saturation had been reached, i.e. no new themes emerged from the analysis.

We used a semi-structured interview protocol which was continuously adapted and expanded according to themes identified during the interviews. The interview protocol comprised six major sections: 1) technology and smartphone use in general, including IT literacy and affinity and use of health-related technology; 2) menstrual app use with a focus on purposes of use and consequences on understandings of menstruation, health and self; 3) body and body image, including perception of menstrual cycles and associated factors; 4) interactions with family, partners, friends and healthcare professionals such as gynecologists concerning menstruation; 5) privacy and security issues arising from menstrual app use and feelings associated with these processes; 6) issues of gender representation when engaging in menstrual tracking. The questions aimed to raise awareness of power relations and (gender) inequalities and to encourage users to reflect on their agency and limitations concerning menstrual app use. For the interviews with former menstrual app users, we included questions about their reasons for stopping and alternative ways of menstrual cycle tracking.

### Data analysis

All the interviews were transcribed verbatim in their original languages. Interviews in German and English were transcribed by the interviewer, while the recordings in Spanish were transcribed by an experienced researcher and colleague of the research team. Subsequently, an inductive content analysis was performed, where coding categories are derived directly from the text data [26]. General reading of the interview transcripts was followed by inductive codification, which was carried out independently by the two authors. The codes and categories identified were contrasted and consensus was reached based on criteria for thematic units of interest. The final categories and sub-categories used for analysis were organized around the following thematic fields: 1) technology use; 2) menstrual app use; 3) menstruation and 4) medicalization. The analysis was supported by the N-VIVO 11 program for qualitative data analysis (QSR International, Melbourne, Australia).

## Results

This section outlines eight characteristics of app-supported menstrual tracking. We describe the users’ motivations and needs driving period-tracking, we examine their experiences and we discuss their responses to (undesired) outcomes. To guarantee anonymity to the participants of our study, all names have been replaced by numbers.

### Tracking menstrual cycle regularities

The main reason for app-supported period-tracking was the wish for a tool to observe and analyze menstrual cycles regularities. All of our interviewees stated that they entered their period dates, and most of them (17) compared past and average cycle lengths with the aim of identifying potential (ir-)regularities:

“And then I look at the graphs, well this doesn’t, rather to … I look at how much time it takes every month, well that, to see if it stays more or less the same.” (Pt. 20, 23 years old).

In general, our participants expected menstrual cycles to be regular and consistent durations were understood as an indicator of menstrual ‘normality’ and overall health:

“If you are menstruating it means you are healthy, doesn’t it? If your body, that is, if, if you are regular, yes there is a health component there which indicates that things are going well. If you don’t have it [menstruation] then there might be problems at the hormonal level, that is, it can be a health indicator that you are ok.” (Pt.14, 32 years old).

When our interviewees identified menstrual cycle irregularities their responses strongly varied depending on their experiences and expectations. For instance, some participants who had experienced late or early periods in the past were relieved when they could identify certain patterns of menstrual cycle irregularities via app-supported tracking:

“…because until then [until using the app] I actually didn’t know if I am super irregular or if it’s not so bad. I always thought I was super irregular. Since I got the app I’ve found out that it actually isn’t that irregular, so, every couple of months it’s very late.” (Pt.5, 37 years old).

However, users who experienced significant discrepancies between app-calculated and actual period starting dates reported strong emotional impacts:

“I will never forget the day when it [menstruation] was 18 days late according to what the app told me. It wasn’t the fault of the app but the fault of my body, but … because also we had exams and the stress” (Pt.19, 23 years old).

As evident from the preceding statement, users experiencing menstrual irregularities (10) tended to hold their body and lifestyle responsible.

### Preparing for upcoming periods

Besides identification of (ir-)regularities, the tracking of menstrual cycle dates predominantly served to prepare for future periods. Avoiding surprises, getting ready for upcoming periods, particularly in terms of provision of menstrual products as well as mental preparation were the most common issues raised by the interviewees. For example, one of the participants pointed out how period-tracking supported the coordination of menstrual cycles and working life:

“Well, I use it [the menstrual app] to check, um, when the next period is due, theoretically. Especially when I’m on my period, well I don’t change my appointments, but in order to know, when I am there, I will take something [menstrual product] with me or I prepare mentally.” (Pt. 1, 29 years old).

Preparation of upcoming periods was not limited to immediate period dates but also served as a more general planning of future events such as vacations, as mentioned by another interviewee:

“The app tells me automatically when I open it, OK your next thing [menstruation] looks like that and that, because of course I don’t only need the, the past ones but also the future ones. That means I can roughly calculate, OK well, at this time, do I get it when I’m on vacation? Will I have it at Christmas?” (Pt.6, 34 years old).

As is evident from the last statement interviewees appreciated the immediacy of the information provided by their menstrual apps.

### Getting to know menstrual cycles and bodies

Another motive for app-supported menstrual tracking raised by several of our participants was the desire to explore their bodies and selves. For instance, one interviewee pointed out how menstrual tracking enabled an improvement in knowledge concerning menstrual cycles, body and self:

“the period, I like to observe it as well as my body, right? The variations, the, well in a sense it is cool because it is a good little tool to get to know yourself a bit better.” (Pt. 17, 31 years old).

Sometimes, app-supported period-tracking helped participants to understand fundamental aspects of menstruation, such as menstrual cycle lengths:

“concerning my menstruation, I had always thought that it comes every month. Until I realized it [the cycle] is 28 days [laughter]. So it’s about the basics here [laughter].” (Pt. 1, 29 years old).

In addition to observation and analysis of menstrual cycle dates and variations, menstrual apps offer a plethora of tracking categories. For instance, the most common apps in our study provide users with four to thirty tracking categories and several dozens of subcategories concerning emotions or corporeal states. Among the most prominent categories used by our participants were emotions (14), ovulation (14), pain (8), sexual activity (7), and vaginal discharge (3). Libido, temperature changes, medication intake, weight, food cravings, insomnia and physical exercise were mentioned by one or two participants each. The wide spectrum of tracking categories was perceived positively by most interviewees and promoted the establishment of novel associations between certain factors and menstrual cycles:

“I didn’t know that there were people who had a certain appetite, for example, sleeping more or were more tired, or felt like something sweet or salty, I didn’t know that, so I’ve learned that with it [menstrual app], that, like that you have more [sexual] desire, so I am, I have always been ignorant concerning those things.” (Pt.23, 21 years old).

In similar ways to this participant, most of the interviewees mentioned that period-tracking led to increased menstrual and bodily awareness and helped them improve their knowledge on menstrual cycles. Increased awareness appeared not only to be connected to the plethora of tracking categories menstrual apps provide but also to be a result of the repeated and long-term engagement that most of our participants reported (Table 1).

### Verifying menstrual experiences and sensations

The documentation and visualization of menstrual cycles through the interfaces of period-tracking apps played an important role in the verification and reassurance of menstrual cycle-associated experiences, sensations and feelings:

“So, if I have the feeling that something [menstrual cycle-associated symptom] happens somehow, then I check whether it’s true and yes. And often it is true, yes.” (Pt.9, 32 years old).

Verification through menstrual tracking specifically concerned sensations and feelings conventionally classified as ‘subjective’ and unquantifiable such as emotions, food cravings and libido:

Interviewer: “And how do you feel when you are ovulating?” - Pt.8: “Well, I notice that I find men more attractive, in general [laughter]. Then, when I look at the app, I think to myself, ah OK, all clear [laughter], yes.” (Pt.8, 36 years old).

Through reassuring participants of their sensations and emotions, app-supported menstrual tracking also appeared to compensate for the lack of recognition participants had received concerning their menstrual cycles:

“No, it is that when I use it [the app] I feel, since it shows me a lot of symptoms that you can have, then I feel a little understood, so to speak” (Pt.24, 18 years old).

### Informing healthcare professionals

The possibility of gaining reassurance with regard to menstrual experiences also influenced our participants’ interactions with healthcare professionals. Several interviewees stated that they used period-tracking apps in order to provide their physicians with menstrual cycle-associated data. A commonly mentioned practice was checking the starting dates of current cycles prior to or during gynecologic visits. Moreover, some participants also observed their menstrual cycles as part of their health management. For instance, a participant who had suffered from a severe illness in the past (Pt. 6), stated to use the app to monitor cycle regularities which served as an indicator for correct medication doses. Another participant, who was prescribed hormonal contraception in order to reduce strong and painful menstruations, pointed out to use the menstrual app in order to provide physicians with exact period dates:

“I use it to know, to enter when exactly the [period] was, because I am taking the pill, so at the doctor I need to tell him exactly the days I have it and it’s for controlling that, for knowing when I had it, and to see if it comes regularly or not.” (Pt. 13, 18 years old).

As indicated by the examples given here, documentation of menstrual cycle dates via menstrual apps seemed to facilitate interactions between users and healthcare providers.

### Tracking health

The majority of our participants perceived period-tracking as a type of health-tracking. For instance, one of the participants reported charting basal body temperatures in order to ensure that they had re-started ovulating after several years of taking the pill:

“I stopped taking the pill, after seven years of taking the pill. And then I wanted to know how my body works, after stopping the pill. And I think it [the app] is awesome for that.” (Pt.7, 28 years old).

Health observation through menstrual tracking was not limited to corporeal indicators such as cycle dates. As most menstrual apps allow for observation of emotions, motivational levels and sociality, several participants stated that they engaged in period tracking in order to know more about the possible connections between cycle-associated (hormonal) changes and mood swings:

“In my case it’s more about the hormones and the moods. And actually, it’s more about ovulation, less about the period, because I already know that I am in a shitty mood when I’m on my period.” (Pt.4, 22 years old).

As is evident from the preceding statement, the tracking of ovulation does not have to be connected to observation of fertility or contraception but can serve other means such as exploration of mental health issues. Nevertheless, some participants did mention tracking their reproductive health via menstrual apps:

“and also, because, because I am thinking of, of having children, not yet but because of my health, as I’ve had also ovarian cysts because of, because of not having the period regularly, so for, for controlling a bit the menstruation.” (Pt.16, 32 years old).

### Contraception and seeking pregnancy

Although several participants reported that they were tracking ovulation, very few of the heterosexual interviewees mentioned relying on the apps’ calculations for contraception. Nevertheless, some stated that they used their apps’ predictions as a guideline:

“It guides me yes, since I am aware of it, of the days that are supposedly fertile and I try to, whatever, if I have relations to be more cautious, to use condoms, this kind of things, right? But yes, I let myself be guided a little by the app.” (Pt.21, 30 years old).

One participant mentioned using a menstrual app primarily as a reminder to insert or remove their contraceptive vaginal ring. Another interviewee stated to use the period-tracking app in order to increase their chances of becoming pregnant:

“I am observing all of that because, as I’ve already told you, I am trying to have another baby, and like that I am way more involved in the whole process, in the whole menstrual cycle.” (Pt.18, 40 years old).

### Changes in tracking, stopping tracking

As is evident from Table 1, most of our interviewees (18) had been tracking their periods for one to several years at the time of the interviews. However, most participants reported on changes regarding the categories they were tracking. For instance, a few of the participants planned to track more categories or collect more exact data in the future:

“I have always thought that I am very irregular but actually I’m quite regular, whereas, um, and I want to have a closer look at that, so I have to collect a few more data.” (Pt.9, 32 years old).

Most interviewees mentioned a decline in the categories they were tracking over time. This was mainly due to changing life circumstances or occurred when participants felt they no longer needed the technology in order to observe their bodies and experiences:

“Well, in the beginning I entered, well you do have the possibility to chart certain emotions. And I used to do that in the beginning. And then I found out that I am in a good mood when ovulating. And that I am in a bad mood the days before the period. I entered that in the beginning until I recognized the rhythm myself, now I don’t have to enter it anymore.” (Pt.7, 28 years old).

Among the 26 participants of our study, three had stopped tracking their menstrual cycles via apps. While one interviewee mentioned having stopped due to amenorrhea (absence of period), the other two users raised privacy issues and pointed out the amount of work required when using menstrual apps:

“…because one needs at least 15 minutes a day to enter everything [into the menstrual app], because one has to read a lot. And then to estimate the severity of one’s symptoms, this requires quite a lot of time (…) it was also strange, because, I mean it is quite intimate (…) and I think it is quite weird that you have to enter that into the internet again. This for sure played a role in stopping it.” (Pt.11, 28 years old).

## Discussion

In public health literature, there are two major strands regarding the implications of health apps. Given that low health literacy is commonly understood to represent a public health problem as it negatively affects people’s access to, understanding and use of health information [27], health apps have often been conceptualized as revolutionary tools for patient empowerment and education (e.g. [28]): Yet, critical accounts have warned of negative consequences such as shifts in responsibilities from health professionals to laypeople and the intensification of body enhancement and healthism imperatives [11, 29].

In recent years, periods have received increasing scholarly attention, and menstrual activism combating related stigma and lack of information has risen [30]. Nevertheless, menstrual cycle-related literacy remains limited among the general population. In 2017, a survey carried out with Austrian adolescents revealed striking knowledge gaps as 17% of the female and 34% of the male participants were unable to define menstruation [31]. Spain is home to Europe’s first menstrual education community and its high number of members reflects the urgent need for spaces for the exchange of period experiences and learning [32]. Therefore, app-supported menstrual tracking might represent a promising tool for user empowerment and for the improvement of menstrual cycle and health literacy. We present a qualitative study conducted with menstrual app users in Europe. Based on qualitative interviews with (former) period-tracking app users, we have identified eight characteristics of app-supported menstrual tracking. Many of the themes emphasize menstrual apps’ potential regarding user empowerment and improvement of menstrual literacy: our participants mentioned being able to better prepare for future periods, using apps in order to support family planning and to verify their experiences and sensations. Moreover, interviewees said that apps helped increased their awareness and understanding of their menstrual cycles and bodies and that they facilitated conversations with healthcare providers. The characteristics mentioned so far confirm the findings of preceding studies highlighting the reasons why people use menstrual, sex, fertility and pregnancy apps: to seek information and to raise awareness [8], to monitor their bodies, to gain reassurance through apps [19] as well as to maintain and establish social relationships [20].

We have also made many findings that reveal a more nuanced picture of the potentials and challenges of app-supported period-tracking. For instance, we describe how the tracking of menstrual cycle (ir-)regularities reinforces associations of regularity and general health. This goes against the findings of social-scientific works [33] as well as biomedical studies [34, 35], which have thoroughly questioned this relationship stating that certain irregularities are common and non-pathological. We show that, depending on participants experiences and expectations, the tracking of cycle (ir-)regularities has a calming effect on some users while leading to distress in others. In this context we also highlight users’ trust in the apps’ representations, and their tendency to blame their bodies and lifestyles for menstrual irregularities. In some cases, menstrual tracking promoted the establishment of novel associations between certain symptoms, experiences and menstrual cycles, thus leading to increased knowledge. For instance, menstrual tracking was used to better understand mental health states by some of the participants. In other cases however, users could be tempted to associate all kinds of symptoms with menstruation, thus overlooking other causes for health issues. Finally, our study also highlights changes in the use of tracking categories used over time and included three participants who had stopped tracking their periods via apps. Whereas a few of the participants wanted to collect more data in the future, many noted a decline in the categories used. We found that reduced tracking was often associated with increased knowledge making some of the apps’ features obsolete. Similar observations have been made by Peng et al. in their study on health apps [8]. Several participants mentioned concerns regarding privacy and security issues and pointed out the workload involved in tracking. For two of the interviewees these issues represented reasons for stopping tracking.

### Practical implications

App-supported tracking of menstrual cycles and associated factors has an impact on people’s experiences and understanding of menstrual cycles, bodies and health, and influences the interactions between health promoters and patients. As demonstrated by our study, experiences vary between users. Here we offer recommendations for healthcare providers and app developers with the aim of tackling potential inequalities that might be reinforced through digital self-tracking practices [11, 36]. Since other types of health apps offer similar features such as provision of information and observation of health-related factors, most of our suggestions also address health apps in general.

### Implications for healthcare providers

#### Integration into healthcare procedures

As demonstrated by our findings, menstrual tracking via apps was often perceived positively as it heightened bodily awareness, improved users’ knowledge on menstrual cycles, and provided them with novel information concerning physiological and psychological states. However, although health apps can have empowering effects concerning users’ personal health management, we want to stress here that app-supported health-tracking should not be perceived as a replacement for personal interactions in the healthcare context. Rather, we recommend a better integration of health apps’ possibilities and challenges into health consultation. Time saved through the ability to observe, analyze and collect health-related information via apps could be used to address and clear possible doubts that might arise. In the case of period-tracking this could include addressing topics such as menstrual irregularities or identification of symptoms associated with menstruation. We suggest that healthcare experts provide the necessary professional and comprehensive support so that all patients are able to take informed and reasoned decisions.

#### Implications for app developers

Our findings also highlight some important characteristics that contribute to the improvement of menstrual app design. The central issues we want to emphasize here are acknowledgement of the apps’ limitations and consideration of the varying profiles, motivations and objectives of people engaging in app-supported menstrual tracking. Our recommendations seek to increase apps’ inclusivity and thus improve their usability and acceptance.

#### Adaptability according to users’ needs

Health app designers should provide the possibility of adjusting the apps’ functions according to user objectives. As pointed out by Epstein and colleagues for the case of menstrual tracking [18], needs for the prediction of menstrual and ovulation dates vary depending on users’ motivations and goals. The authors state that women tracking their cycles in order to become pregnant might appreciate conservative estimates of their fertile window while women using menstrual apps as a contraceptive method are likely to prefer overestimations. Drawing on our findings, we want to add here that the tracking of ovulation is not necessarily connected to reproduction. In order not to reproduce heteronormative assumptions, we urge designers to reconsider the naming of the days surrounding ovulation and offer users the possibility to independently track ovulation and fertile windows. Gender-sensitive approaches with regard to app functions and nomenclature are reasonable for other health apps as well, especially concerning those addressing either male or female users. Furthermore, we suggest that designers should provide menstrual app users with flexible rather than definite predictions of menstruation dates in order to avoid negative experiences. For instance, apps could offer their users a wider range of potential future period dates. To our knowledge, this suggestion is indeed met by one of the apps used by our interviewees by displaying a graphic representation instead of a numerical countdown on the home screen. An alternative would be the creation of interfaces that present probabilities as suggested by Epstein et al. [18].

#### Acknowledgement of limitations

In order to avoid emotional distress, health apps should provide users with information on their limitations. For instance, in addition to offering more flexible depictions of menstruation and ovulation dates, period-tracking apps could inform their users that irregular periods are common and not necessarily an indicator of bad health [34, 35]. Given the wide range of tracking categories offered by menstrual apps, we believe that apps should inform their users that certain factors might be but are not exclusively related to menstrual cycles in order to avoid further medicalization of menstrual cycles or the drawing of wrong conclusions. Regarding health apps in general, developers should offer users the option of selecting the categories they want to track in order to make interfaces clearer and avoid additional, undesired workload. Concerning the privacy issues that have been mentioned by some participants, we urge designers to inform users in a comprehensible manner about the kinds of data that are being collected including the reasons for collection and planned or potential usages.

#### Limitations

This study has been developed with a bias toward understanding certain topics of interest, foregrounding issues identified by former studies on health-related self-tracking. Thus, it is not representative of any particular population or specific type of menstrual app, but rather offers first impressions and insights into menstrual tracking practices, experiences and consequences. Future studies including mixed-method approaches are necessary to get insight into general patterns of menstrual app use and its implications, for instance regarding social inequalities. Although the researchers sought to include varying individual profiles, the final demographic makeup of the participants was strongly influenced by the researchers’ networks (academic, White, middle class). Since the two researchers are from different countries, differences in language skills and knowledge of cultural specificities represented further obstacles in the present study. In order to minimize the difficulties, interview transcripts in German were translated to English and all transcripts were read repeatedly line-by-line and cross-checked by the two researchers.

## Conclusions

This qualitative study gives insight into people’s practices and experiences of app-supported menstrual tracking and reports on its potentials and challenges regarding user empowerment and improvement of menstrual and health literacy. The results inform researchers, app designers, and health care providers about users’ experiences and consequences of app-supported period-tracking. Moreover, the study provides suggestions for the integration of self-tracking technologies into existing healthcare procedures and for further development of menstrual app design. In order to obtain a more complete picture regarding the role of tracking technologies within the healthcare context, future research should incorporate participants with more varied social backgrounds such as people with limited health and digital literacy. Additionally, researchers should also investigate app developers’ perceptions and objectives, and provide insight into healthcare professionals’ opinions and experiences concerning the consequences of health app use on patient-doctor interactions.

## Data Availability

The dataset supporting the conclusions of this article will not be shared publicly, due to information contained within the interviews that could be linked to participants.
